# Obsessed

**Published:** 1914-07

**Authors:** 


### Obsessed.

It could be stated, without fear of serious contradiction, that society is becoming obsessed with the theme of sex. This is made evident by the character and large output of our current literature. Judging from the signs of the times, our activities and interests are all being permeated with sex ideas. The followers of Freud believe that everything fundamental in life is attributed to sex, and that life itself is one long sex pursuit. It matters not what our actions are, or what we see in others or what we read, or hear, there is the same conscious or unconscious suggestion to some phase of sex.

The effect of this psychological obsession on society is very apparent. We are fast developing a new "mores," a new (moral) code, the ultimate result of which is not hard to conjecture. We have heard much of the "conspiracy of silence." But with all its baneful influence there is less to be feared than from this wild rush of freedom. When every motive or action, when every word or expression, when every ill or affection has ascribed to it some expression of sex, then, surely there is something radically wrong. Sex, like any other normal function of the body, is natural, and therefore is not unmoral. And, like any other normal function of the body, it is made abnormal by abuse or lack of proper stimulus. We may grant that it is the strongest and most insistent of all the instincts, but it is only a part of the whole and, if developed out of proportion to its normal relation, it will dominate all the rest. It is subject to the will and may be refined by education, and it serves him best who dispenses culture with vocation and passion with temperance. The normal human life is a carefully adjusted balance between its various activities. Every function has its exciting and inhibitory stimulus for the purpose of maintaining this adjustment.

The sex instinct is no exception to the rule, and the trouble now is that this balance has been lost. The inhibitory stimulus, like modesty, cultured manners, etc., has been measurably destroyed. While the exciting stimulus like dress, the modern stage and literature, and loss of reserve has been unbridled.

The *British Medical Journal,* as quoted in the *Current Opinion,* gives a very lucid explanation of the psychology of this obsession in controverting the Freudian ideas:

"Is it not possible," asks the author, "that the explanation of such a result is to be found in a conception of the mind based upon the structure of the central nervous system, rather than in the view that the sexual feelings are always struggling to the surface, that they are kept in a supposed state of 'repression' by a fictitious 'psychic censor'?

"The whole of the nervous system is regarded as built up of an immense number of sensori-motor arcs, tracks along which impulses for sensation and motion travel. The more complicated the life of the individual, the more numerous and complex the paths which are laid down. Certain tracks used habitually become pushed out of their connection with consciousness. They have to do with reflex and automatic activities. The tracks which have to do with more difficult and complicated reactions are on a higher plane and impulses passing through them obtrude themselves into consciousness. But the connection between the tracks are numerous and irritation applied at one end of the track, to which the response is simple in normal conditions, may overflow into other neighboring tracks.

"Similarly with the mind, the sexual path being of enormous importance to the race is very open, and easily allows influences from other paths to flow into it. But this does not mean that it is perpetually struggling into consciousness. Sexual gratification gives rise to a feeling of satisfaction; but this is not the same as saying that all feelings of satisfaction have a sexual basis. To pour out one's woes to another and obtain his sympathy gives a feeling of satisfaction. To say that the friendships felt as a result of this sympathetic attitude is due to sexual *libido* is preposterous. In certain perverted individuals it is conceivable that the impulse passing through the reflex arc of sympathy at the sight of a friend may overflow into the ever-ready sexual arc, and lead to homosexuality. But there is not the slightest reason to write as if homesexuality were the natural result of friendship; rather it would seem that friendship in the abnormal may lead to homosexuality."

